# Improving Osteoporosis Management in Primary Care: An Audit of the Impact of a Community Based Fracture Liaison Nurse

**DOI:** 10.1371/journal.pone.0132146

**Published:** 2015-08-27

**Authors:** Tom Chan, Simon de Lusignan, Alun Cooper, Mary Elliott

**Affiliations:** 1 Clinical Informatics, Department of Health Care Policy and Management, University of Surrey, Guildford, Surrey, United Kingdom; 2 Bridge Medical Centre, Three Bridges Road, Crawley, West Sussex, United Kingdom; David Geffen School of Medicine, UNITED STATES

## Abstract

**Background:**

Osteoporosis and associated fragility fractures are a major health problem; they are more common in women over 50 years old. Fracture liaison nurses have been widely used in secondary care to promote the recognition of fragility fractures and to promote the use of bone-sparing medication to reduce the risk of recurrent facture.

**Objective:**

Audit the impact of a primary care based fracture liaison nurse on the detection of fragility fractures in people with osteoporosis and their treatment with a bone-sparing medication.

**Method:**

This audit took place in 12 GP practices using ‘before and after’ cross-sectional extractions of anonymised routine data. We report, for females 50–74 years and ≥75 years old, socio-economic deprivation index, the prevalence of osteoporosis, recording of fragility fractures, dual-energy X-ray absorptiometry (DXA), smoking, and body-mass index (BMI) and use of appropriate bone-sparing medication. We used Altman’s test of independent proportions to compare before and after data.

**Results:**

Recording of the diagnosis of osteoporosis increased from 1.5% to 1.7% (p = 0.059); the rate of DXA scans fell (1.8% to 1.4%; p = 0.002); recording of fractures and fragility fractures more than doubled (0.8% to 2.0%; p<0.001 and 0.5% to 1.5%; p<0.001, respectively) with approximate doubling of the recording of smoking, and BMI (p<0.001 level). Fragility fracture recording rose from 8.8% to 15% in females aged 50 to 74, and from 0.8% to 2.3% in people aged ≥75years old (p<0.001). There appeared to be inequity in the service, people who were least deprived were more likely to receive DXA scans and the more deprived to be prescribed bone sparing agents.

**Conclusion:**

A fracture liaison nurse in primary care has been associated with a period of improved management. Liaison nurses based in different parts of the health system should be tested in a prospective trial.

## Introduction

Osteoporosis and associated fragility fractures are a major health problem; and fracture liaison services, usually based in secondary care, have been established to try to ensure that people with these fractures receive appropriate therapy.[1, 2, 3]. Fragility fractures are more common in women over 50 years old; and despite the development of a number of evidence-based therapeutic interventions and quality improvement initiatives there remain gaps in the management of osteoporosis. Adverse socio-economic status is also strongly associated with higher bone turnover [4]. The bisphosphonates are probably the most important bone-sparing agent; they are known to be effective in managing osteoporosis and reducing the rate of re-fracture but gastrointestinal side effects and other problems means that adherence is poor. Fracture liaison nurses have been widely used in secondary care to promote the recognition of fragility fractures and to promote the use of bone-sparing medication, principally bisphosphonates, to reduce the risk of recurrent facture [5, 6].

Comprehensive computerised primary care records have the potential to identify fragility fractures, cases of osteoporosis, and its risk factors; with the potential to provide an osteoporosis liaison service a holistic view of risk factors and adherence to therapy, and audit the equity of the service. The UK has a registration based primary health care system: one patient can only register with one general practice, and all patients have a unique ID which links their all their medical records, NHS number. A strength of this system is that primary care records can be used to assess equity of service provision; and to confirm or refute the equity of testing [7]^,^ or treatment [8].

Primary care computer records are generally made at the point of care, with records containing information about each encounter [9, 10]. A pay-for-performance (P4P) system to improve chronic disease management, introduced in 2004 further improved recording but also has distorted some records [11]. Routine recording of cases of osteoporosis, fragility fractures and risk factors has been possible for some time [12]; though data quality has been variable [13, 14]. The advantage of primary care records over those in secondary care should be the more comprehensive availability of data about comorbidities (e.g. rheumatoid arthritis) or use of steroids [15]; and osteoporosis mimicking conditions [16]. It is also possible to explore prescriptions as a proxy for adherence Osteoporosis has been introduced into the P4P system 2012.

We hypothesised that the introduction of a community based fracture liaison nurse would improve the detection of fragility fractures in people with osteoporosis and their treatment with a bone-sparing medication. We planned to test the hypothesis by analysing the study practices’ performance against the emerging “Quality and Outcomes Framework” (QOF) pay-for-performance indicators using a before and after method. We defined a case for inclusion as female patients, ≥50 years old with a coded diagnosis of osteoporosis or an associated operation; or a computer record of treatment only used in osteoporosis. The intervention was the introduction of a community based fracture liaison nurse to the study general practices. The fracture liaison nurse reviewed the practice population who met our inclusion criteria. Our analysis included the following variables associated with the development and evidence of osteoporosis: age, gender, therapy, body mass index (BMI) [17] smoking status [18], socio-economic deprivation [4], bone sparring therapy, vitamin D level recording, record of fragility fracture and having had a bone density scan (strictly a dual-energy X-ray absorptiometry (DXA) scan).

## Method

### Overview

We report an audit of care of people with osteoporosis, carried out in twelve practices in the southeast of England, with a combined registered population of 120, 009 people. The data for this audit were collected in 2010; before and after period are defined as the 12 months before 01/04/2009 and 12 months after 01/04/2009, the date that the primary care fracture liaison nurse started.

Consent for data collection was obtained from individual practices. Each practice was sent a letter explaining the study, the practice’s involvement and the process of the data extraction.

Pseudonymised data for the female practice population 50 years old and over were extracted from the consented GP practice databases using MIQUEST (Morbidity Information Query and Export Syntax), an NHS sponsored programme which enables the extraction of the same dataset from different brands of GP electronic patient record (EPR) system by trained data collectors who receive information governance training and the data was processed using a standardised method [19]. The post codes were converted into index of multiple deprivation [IMD] at source, so that no identifiable data left the practice. The IMD, is a National Statistics developed measure that can be used to divide the population into ten equal deciles from most to least deprived [20].

The criteria included the audit were females in a high risk age group (≥50years old). The disease register was defined by the use of an appropriate computer code for a fractures including likely fragility fractures.

For this group we also extracted data about:

1. Important modifiable risk factors: Body Mass Index (BMI) and smoking;
2. Investigation by DXA scan and
3. Treatment of high risk individuals with bone-sparing therapy, principally bisphosphonates.

After the baseline audit the standard aimed for was to achieve compliance with anticipated pay-for-performance (P4P) indicators due to be introduced into primary care [21]. We completed the audit cycle by comparing before and after cross-sectional extractions of anonymised routine data. We compared two cross-sectional data samples; making before and after comparisons of the originally set audit criteria; and later added conformance with the proposed osteoporosis P4P indicators.

### Subjects and setting

The study took place in practices who had agreed to join a community fracture liaison nurse pilot. This was not mandated and practices could have participated in the pilot but not the audit, however they all opted in. The subjects were females age 50 years or older.

### Pay-for-performance (P4P)

Our analysis was carried out at a time when pay for performance (P4P) was being introduced for osteoporosis. This was a powerful motivator for practices to participate, and may explain why all the eligible practices participated. The P4P was called a “Directed Enhanced Service (DES), which included an scheme to promote treatment of postmenopausal women [22], highlighting the need for enhanced monitoring of the disease in general practice. The osteoporosis DES was influenced by guidance available at the time: (1) The World Health Organization (WHO)-supported approach to the assessment of fracture probability [23]; (2) The creation of UK consensus guidelines for the management of osteoporosis [24]. The major national primary care P4P scheme, the “Quality and Outcomes Framework (QOF)” [25, 26], which had been introduced in UK primary care in 2004, also planned to include osteoporosis.

### Population at risk

The audit focussed on females 50-74years, and ≥75years old, on the basis that the first, younger, group who have fragility fractures should have a DXA scan and bone sparing therapy; whilst the older group with a fragility fracture should just receive treatment. Within these age-bands people were identified as having osteoporosis on the basis of a disease code or treatment with a therapy (e.g. bisphosphonate) only used in osteoporosis. We used our ontologically rich approach to identifying variables, identifying a range of different codes that might represent a clinical concept [27].

The audit also flagged that people in residential homes may benefit from receiving vitamin D, or calcium and vitamin D supplements [28]. This benefit may pertain whether or not they have a low level [29]. Guidance from the National Osteoporosis Society (NOS) recommends vitamin D supplements where people are primarily indoors and exposed to low levels of sunlight [30] Poor appetite and diet may exacerbate this situation further. However, the National Institute for Health and Care Excellence (NICE) is more guarded and whilst acknowledging there is evidence of benefit stops short of making this recommendation [31].

### Fragility fractures

A fracture record was a derived variable that is indicated where there is a record of fracture diagnosis, or the presence of a record of operations for reduction and/or fixation of fracture suggestive that it was a fragility fracture (e.g. Read code 7K23-4 Austin Moore hemiarthroplasty would imply a fragility fracture of hip; Read code 7K1LL–Closed reduction of fracture of radius and/or ulna, including Colles we would interpret as a fragility fracture of the wrist.) We extracted data about DXA scans.

### Reversible risk factors and therapy

We also extracted data about smoking and BMI, as potentially reversible risk factors. Both a low BMI <20kg/m^2^ or a high BMI >30 Kg/m^2^ are associated with increased risk. We also extracted data about the prescription of calcium and vitamin D preparations and bisphosphonates.

Association of deprivation with fragility fractures, DXA scans and use of bone sparing agents

We hypothesised that, in common with much multi-morbidity [32], that fragility fractures might be more common with increased deprivation and that DXA scans and bone sparing agents might be more common among the less deprived.

### Data processing

We only extracted coded data from practice systems, not free-text, using well established processes to ensure patient privacy [33]. Our extraction method also took account of potential data extraction errors,[34] and the different coding systems used within the locality [35]. We processed these data using methods we have developed over the last 16 years [36, 37].

### Statistical methods

We used descriptive statistics to describe findings; we used Pearson Chi square to compare whether proportions are significantly different; Spearman’s rank sum text to compare non-normally distributed trends; and Altman’s test of independent proportions to compare before and after data [38].

### Ethics statement

The study fulfils the Health Research Authority, National Research Ethics Service definition of an audit, and therefore did not require research ethics review [39]. This audit was approved by the all the participating practices based in Crawley.

## Results

### Audit population and prevalence of osteoporosis

**Fig 1:**
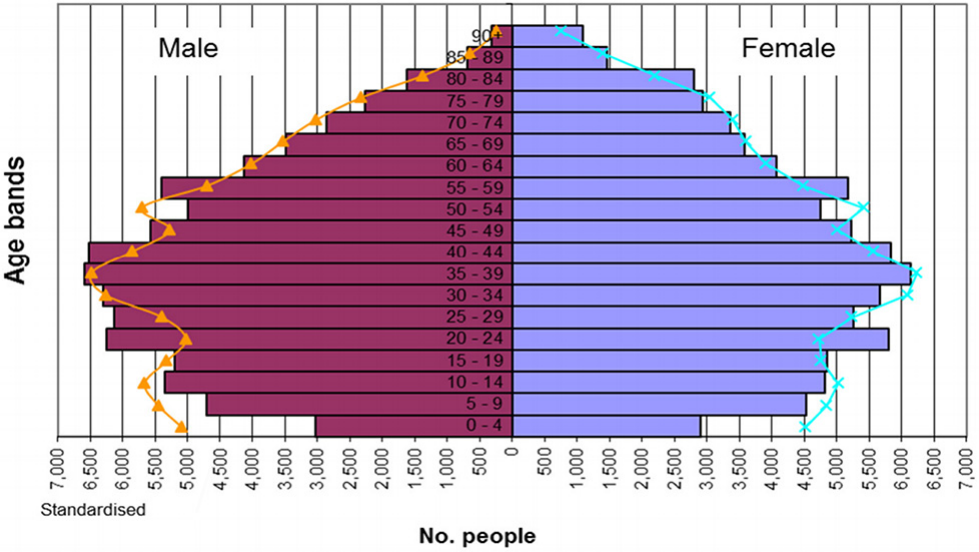
Age-sex pyramid of the practice population, the age-sex profile of the practices is similar to the national average.

The study population had an age-sex profile close to the national average (census 2001 [40]); 52.3% (n = 19,796) of women are aged ≥50 years (Fig 1). There are fewer young children (age under 5 years) and an excess of adults in their 20s in the population; but these differences have no impact on the study.

There was no significant change in the population as a whole, between the study before period (01/04/2008 to 01/04/2009) and after period (01/04/2009 to 01/04/2010), nor in people age 75years or over.

Osteoporosis was more common with increasing age in the participating practices. All but one of the practices had approximately doubled the prevalence of osteoporosis in women aged ≥75years, compared with the 50 to 75years old age-band, at the baseline data collection this was 6.7% vs 3.4% (Table 1).

**Table 1:** Baseline recording of a diagnosis of osteoporosis in audit practises, recorded prevalence in the ≥75years group compared with the 50 to 74year old age group.

	Age 50 to 74 years			Age 75 years and over		
	Population	Diagnosis of Osteoporosis		Population	Diagnosis of Osteoporosis	
	N	n	%	N	n	%
Practice 1	1171	32	2.7%	338	17	5.0%
Practice 2	1868	58	3.1%	547	32	5.9%
Practice 3	1294	44	3.4%	337	29	8.6%
Practice 4	868	24	2.8%	277	14	5.1%
Practice 5	1332	72	5.4%	432	48	11.1%
Practice 6	1261	30	2.4%	341	16	4.7%
Practice 7	2193	92	4.2%	679	49	7.2%
Practice 8	1075	42	3.9%	362	26	7.2%
Practice 9	687	25	3.6%	213	15	7.0%
Practice 10	573	8	1.4%	85	1	1.2%
Practice 11	967	27	2.8%	214	12	5.6%
Practice 12	1231	39	3.2%	332	21	6.3%
**Population**	**14520**	**493**	**3.4%**	**4157**	**280**	**6.7%**

**Fig 2:**
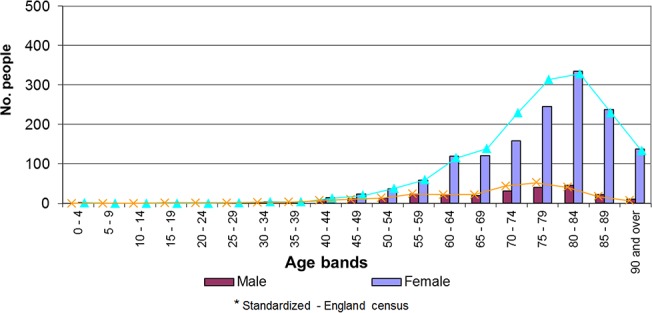
Standardised age-sex profile for people with a diagnosis of osteoporosis.

There was a statistically significant correlation between proportion of practice population aged ≥50 and proportion of patients with a record of fracture (Spearman’s rank correlation 0.582; 2 tailed sig p = 0.037). The standardised prevalence of osteoporosis rose from 1% in females aged 50-54years old, through to 13% in females aged 70-74years. It rose to 17% in females aged 75-79years and by 1–2% further for each subsequent 5-year age band (Fig 2).

**Fig 3:**
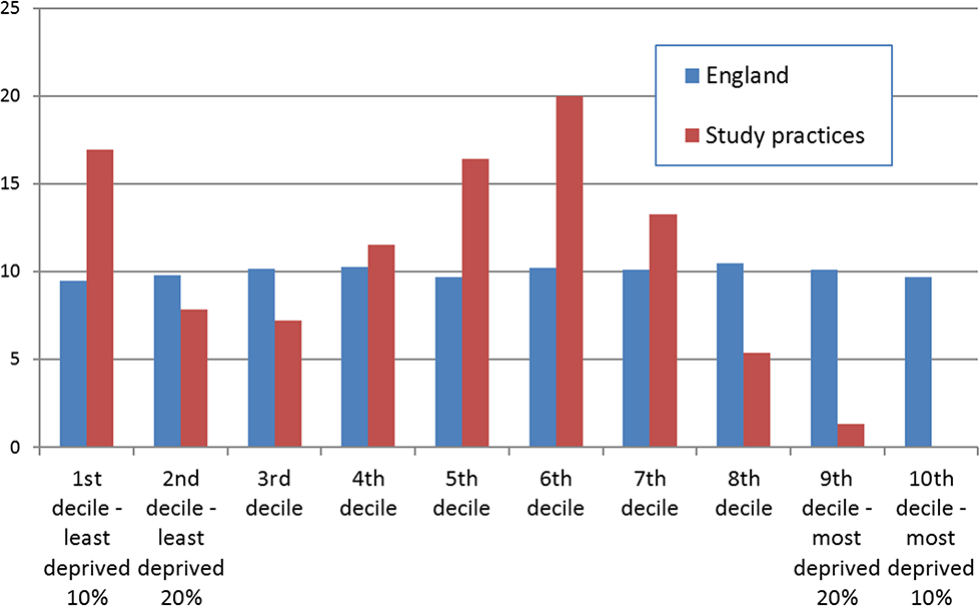
Indices of multiple deprivation (IMD) for the audit practices combined population, the population is less deprived than for England in general but has a bimodal distribution with first, fifth, six and seventh above the national average.

The study population was found to be less deprived than the English average with a greater proportion of people in the 1^st^ (least deprived), 5^th^, 6^th^ and 7^th^ deciles; with a lower proportion of people in the 2^nd^, 3^rd^, and 8^th^ deciles. There were very few people in the 9^th^ and nobody in the 10^th^ (most deprived) decile (Fig 3).

### Fractures

Recording of fractures, smoking and BMI rose significantly in the course of the audit. Fracture recording was three times as high in females 75years and over compared to those who were 50 to 74years old (Table 2). The recording of any fracture and of fragility fractures rose from 0.3% to 0.9% (p<0.001) and 2.2% to 4.8%; (p<0.001), before and after in the 50-74years old and 75years and over age groups. Fragility fracture recording rose from 0.2% to 0.8% in females aged 50 to 74, and from 1.2% to 3.4% in people aged ≥75years old.

**Table 2:** Recording of key audit variables before and after the ‘intervention’ for all registered patients, females 50 to 74years old, and females 75years old or older.

Practice Population	Before		After		*p
	n	%	n	%	
Has record of Osteoporosis diagnosis	338	0.3	430	0.3	0.514
Has record of fragility fracture	94	0.1	327	0.3	0.000
Has record of fracture	238	0.2	548	0.4	0.000
Has record of DEXA	431	0.4	405	0.3	0.000
Has record of bone sparring agent	1440	1.4	2013	1.6	0.000
Has smoking status record	27409	27.0	44347	36.1	0.000
Has BMI record	17983	17.7	33415	27.2	0.000
**Denominator population**	**101649**	**100.0**	**123009**	**100.0**	
**Female age 50 to 74years**	**Before**		**After**		*p =
	**n**	**%**	**n**	**%**	
Has record of Osteoporosis diagnosis	93	0.7	159	1.1	0.000
Has record of fragility fracture	26	0.2	114	0.8	0.000
Has record of fracture	47	0.3	136	0.9	0.000
Has record of DEXA	211	1.6	204	1.4	0.000
Has record of bone sparring agent	367	2.7	556	3.8	0.000
Has smoking status record	4155	30.7	6646	45.8	0.000
Has BMI record	2574	1.9	5413	37.3	0.000
**Denominator population**	**13519**	**100.0**	**14520**	**100.0**	
**Female aged 75years and over**	**Before**		**After**		*p =
	**n =**	**%**	**n =**	**%**	
Has record of Osteoporosis diagnosis	181	3.7	185	3.5	0.626
Has record of fragility fracture	58	1.2	180	3.4	0.000
Has record of fracture	108	2.2	254	4.8	0.000
Has record of DEXA	121	2.5	75	1.4	0.000
Has record of bone sparring agent	809	16.6	1077	20.4	0.000
Has smoking status record	1458	29.9	2808	53.2	0.000
Has BMI record	1023	21.0	2132	40.4	0.000
**Denominator population**	**4883**	**100.0**	**5276**	**100.0**	0.626

*Altman’s test of 2 independent proportions was used to calculate the p value.

### Reversible risk factors

There was a highly significant increase in the recording of smoking by at least half across the practice population and the high risk groups. The increase was greatest in women aged ≥75year old. There was approximately a doubling of the recording of BMI (changes all significant at p<0.001 level).

### Dual-energy X-ray absorptiometry

The rate of DXA scans fell (2.5% to 1.4%; p-0.002); though (p<0.001). This may have been due to more selective use of scans, as the audit standards set out that scans were not required in people ≥75years old. Also, people involved in the audit reported that they were more likely to code a diagnosis of osteoporosis into their GP computer system rather than code the individual DXA scan results, which tended to report multiple findings. DXA scans often included multiple results from spine, hip and wrist and were complex to code; and that some GP computer systems would not accept negative numeric values making it difficult for diagnostic t-scores (less than -2.5 standard deviations) to be recorded [13].

### Treatment

Vit D preparations are more likely to be prescribed to people in residential homes; 19.4% (40/206) are prescribed these preparations compared with 3.5% of the general population (p<0.001). The use of bone sparing agents was more likely after the intervention than before; this changed from 1.4% (1440/10649) before to 1.6% after (p<0.001) (Table 2).

**Fig 4:**
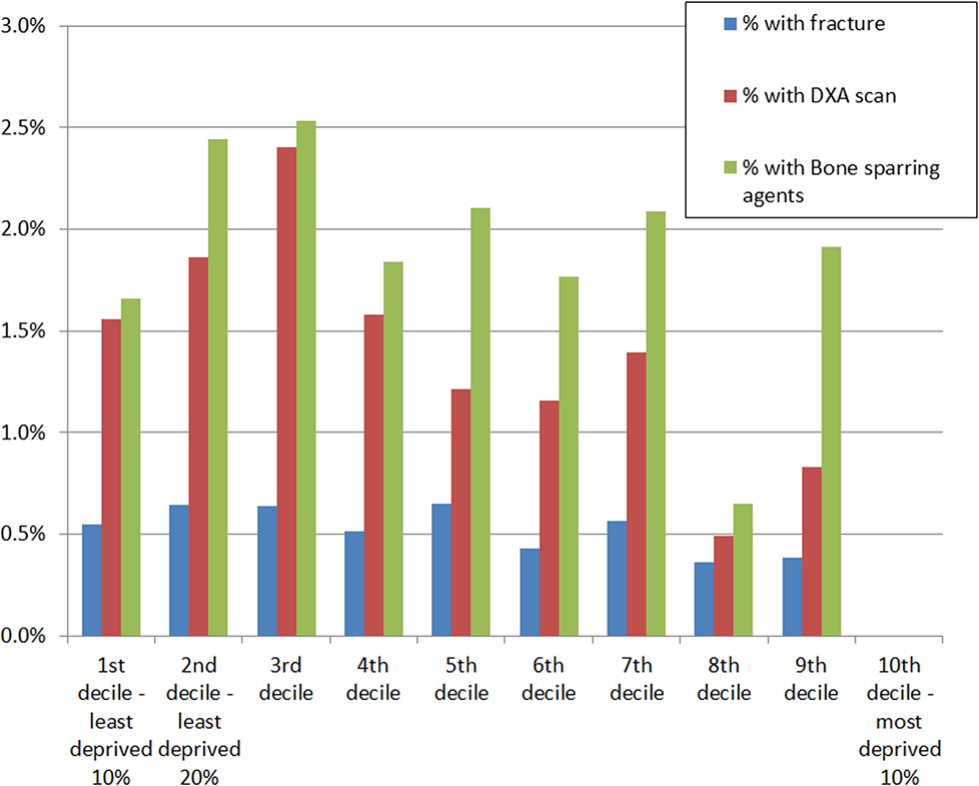
Proportion of people by deprivation decile with fractures, DXA scans performed, and prescribed bone sparing agents.

Association of deprivation with fragility fractures, DXA scans and use of bone sparing agents:

We did not find an increased rate of fragility fractures in the more deprived population. There was a non-statistically significant trend towards less fragility fractures in the more deprived deciles. A greater proportion of people in the top three deciles (i.e. the least deprived third) received DXA scans, and received bone sparing agents (Fig 4).

**Fig 5:**
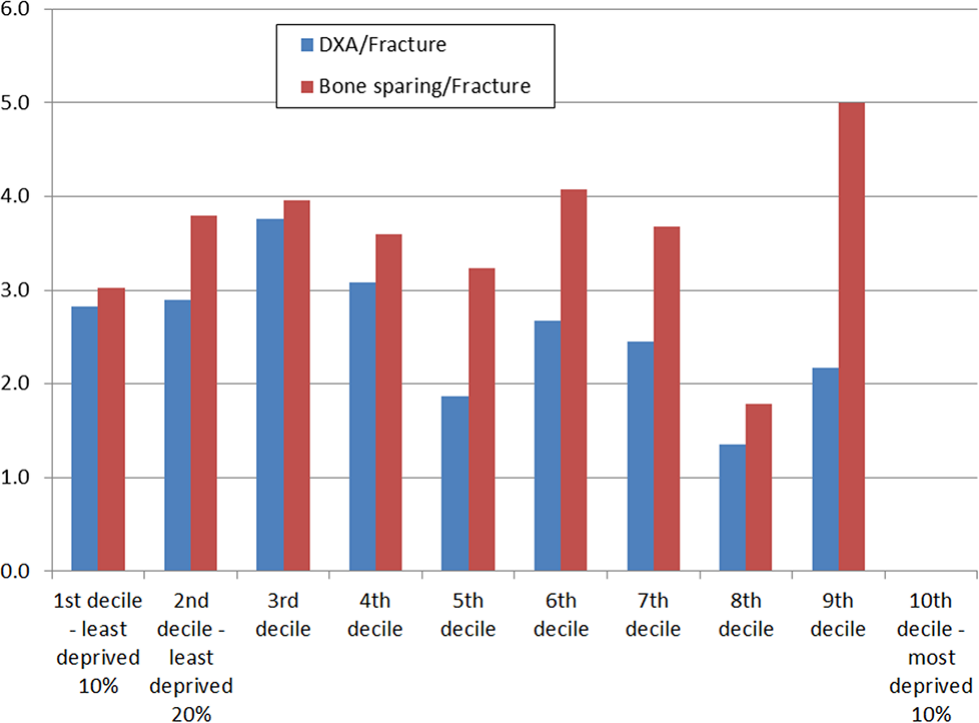
Ratio of DXA scans and bone-sparing prescriptions to fracture rate.

However, when we explored the ratio of DXA scans and people on treatment to the rate of fractures, it appeared that people in the least deprived centiles received more scans but less bone sparing treatment that the more deprived population (Fig 5).

### Specific audit outcomes

The final audit outcomes involved looking at the proportion of people with fragility fracture and a diagnosis of osteoporosis in two age groups 50 to 74years and ≥75years old (Table 3). There was an increase in cases recorded and increased prescribing of bone sparing medications, they rose from 47.9% to 84.0%, and from 39.5% to 70.5% in the 50 to 74years and ≥75years old age groups respectively (p<0.001).

**Table 3:** Audit outcomes, influenced by emerging pay-for-performance indicators.

**Osteoporosis indicator 1(1):**					
**The practice can produce a register of patients: 1. Aged 50–74 years with a record of a fragility fracture**					
	**Before**		**After**		
	**n**	**%**	**n**	**%**	***p =***
Aged 50–74 years	15	8.8%	25	14%	= 0.168
Denominator population	171	100%	178	100%	
**Osteoporosis indicator 1(1):**					
**The practice can produce a register of patients: 1. Aged ≥75 years with a record of a fragility fracture**					
	**Before**		**After**		
	**n**	**%**	**n**	**%**	**p =**
Aged 75 years and over	65	0.8%	199	2.3%	0.000
Denominator population	8110	100%	8702	100%	
**Osteoporosis indicator 2:**					
**Patients aged between 50 and 74 years, with a fragility fracture, in whom osteoporosis confirmed who are currently treated with an appropriate bone-sparing agent**					
	**Before**		**After**		
	**n**	**%**	**n**	**%**	**p =**
Aged 50–74 years	23	47.9%	42	84.0%	0.000
Denominator population	48	100%	50	100%	
**Osteoporosis indicator 3:**					
**The percentage of patients aged 75 years and over with a fragility fracture, who are currently treated with an appropriate bone-sparing agent**					
	**Before**		**After**		
	**n**	**%**	**n**	**%**	**p =**
Aged 75 years and over	118	39.5	222	70.5%	0.000
Denominator population	181	100%	316	100%	

## Discussion

### Principal findings

The appointment of a primary care based fracture liaison nurse was associated with improved data quality, for diagnosis, fractures, modifiable risk factors and therapy within a year. There was improvement in all areas–more recording of diagnoses, recording of potentially reversible risk factors and treatment. Therapy appeared to be more selectively targeted on people with fragility factures.

However, the study revealed further scope for improvement; and also interesting data about possible disparities. There appeared to be trend to DXA scan more people who are less deprived, and prescribe more bone sparing agents in people from more deprived groups.

### Implications of the findings

The appointment of a primary care fracture liaison nurse appears to have been associated with quality improvement. A practice based fracture liaison nurse would also have access to information not available to hospital colleagues, and beyond the scope of this audit, such as whether a patient is collecting their repeat prescriptions for bone sparing agents, previous prescription or steroids, or other previous important comorbidities. However, it is difficult to know the extent to which there is a causal relationship and the extent to which care might improve, or the cost effectiveness of the intervention.

The population perspective of a community based nurse may enable equity of care to be addressed in a way that would not be possible in the hospital setting.

### Comparison with the literature

The National Institute for Health and Clinical Excellence (NICE) reports the prevalence of osteoporosis in women, increases markedly with age after the menopause, from approximately two per cent at 50 years, rising to more than 25 per cent at 80 years. This suggest that the prevalence of osteoporosis was under-reported in our audit we found 1% at 50years rising to 22% at age 90. The NICE technology appraisal reports 11 per cent of post-menopausal women aged 50 years and over with osteoporosis had a clinically apparent osteoporotic fragility fracture, rising to 19 per cent for ages 65 years and over [41]. Fracture rates recorded in this study are much, much lower.

The age bands used within the audit to determine treatment, principally treating women over 75years old with a fragility fractures with bone sparking agents without a DXA scan were subsequently adopted by NICE [42].

The need for better coordination and integration of care have been flagged by others, however the use elsewhere of a primary care based fracture liaison nurse is novel and not reported within these reviews [43, 44] There may be other factors such as a good diet and exercise, which have been outside the scope of this audit; but might be important in fracture management [45].

### Limitations of the audit

Studies on routine data inevitably have limitations, and whilst many of these are known [46, 47, 48] the restriction to working with coded data in primary care records means that we inevitably under-report some aspects of care, notably DXA scans or other information which might be contained in hospital letters.

The introduction of a voluntary element of P4P in 2008/9 may have influenced the audit, however it would have affected both the before and after year; the rewards for participating in the osteoporosis scheme were relatively small around £600 per practice per year [49], compared with GP income estimated in this period to be in excess of >£100,000 per year [50].

The bimodal distribution of the deprivation index, and the lack of people in the most deprived decile limits the strengths of our observation about deprivation and fragility fractures. However, a previous study has shown lower rates of hip fracture with increased deprivation, with these differences put down to differences in age between groups [51]. Though a previous study had suggested that people from more deprived groups were more likely to have a lower bone mineral density, with the only clearly difference in risk factors that hey smoked more [52].

### Call for further research

Prospective studies are needed to determine whether fracture liaison nurses are best located in osteoporosis clinics, where advice at the time of fracture may have greater impact on patients [53] or in primary care where adherence and other risk factors can be more readily determined [54]. Such studies should also include an economic evaluation.

## Conclusion

A fracture liaison nurse in primary care has been associated with a period of improved management. A community based nurse can also be provided with data to give a population perspective and information about the equity of service provision. Liaison nurses in primary care may be better placed than those in hospital to ensure the implementation of best practice.
